# Estimating Risk of *C. difficile* Transmission from PCR Positive but Cytotoxin Negative Cases

**DOI:** 10.1371/journal.pone.0088262

**Published:** 2014-02-11

**Authors:** Mini Kamboj, N. Esther Babady, Jane W. Marsh, Jessica L. Schlackman, Crystal Son, Janet Sun, Janet Eagan, Yi-Wei Tang, Kent Sepkowitz

**Affiliations:** 1 Infection Control Program, Memorial Sloan-Kettering Cancer Center, New York, New York, United States of America; 2 Infectious Diseases Service, Department of Medicine, Memorial Sloan-Kettering Cancer Center, New York, New York, United States of America; 3 Clinical Microbiology Service, Department of Laboratory Medicine, Memorial Sloan-Kettering Cancer Center, New York, New York, United States of America; 4 Division of Infectious Diseases, University of Pittsburgh, Pittsburgh, Pennsylvania, United States of America; Charité, Campus Benjamin Franklin, Germany

## Abstract

**Background:**

The use of molecular methods to diagnose *Clostridium difficile* infection (CDI) has improved diagnostic yield compared to conventional methods. However, PCR testing can detect colonization and has introduced several practical challenges pertaining to need for treatment and isolation of cases.

**Methods:**

For all new cases detected by real-time PCR, concurrent cytotoxin assay was performed and genetic characterization with MLVA (multi-locus variable number tandem repeat analysis) was done to determine relatedness. We used PCR cycle threshold (Ct) of detection as surrogate marker for bacterial burden in stool.

**Results:**

Overall, 54 cases of CDI were detected during the study period. 42 were concurrently tested by CYT and characterized by MLVA .MLVA analysis revealed marked genetic diversity with no ongoing outbreaks; four cases were due to NAP1 strain. CYT −/PCR + cases had a higher median Ct value of detection compared to CYT+/PCR + cases (28.2 vs 22.5; p = 0.01). Among 25 strains that were genetically related, 9/11 isolates in this dominant cluster were positive by CYT compared to 4/14 in non-dominant clusters (p = 0.02).

**Conclusion:**

CYT−/PCR+ cases contribute to hospital based transmission. However, the risk of transmission of *C. difficile* from CYT +/PCR+ cases may be higher than those that are CYT−/PCR+.

## Introduction

The introduction of molecular diagnostics into routine hospital care has brought remarkable accuracy and speed into the identification of numerous infections.

However, the increased sensitivity of molecular tests has identified many patients, whose infection would have escaped detection utilizing conventional methods, creating uncertainty about when, and for how long to isolate. Of particular concern is *Clostridium difficile*, the most common cause of hospital-acquired diarrhea. At many facilities, the overall detection of *C. difficile* associated diarrhea has increased by 50% or more due to improved sensitivity and favorable operation characteristics of the molecular test [1]–[3]. Yet the contagiousness of patients who are positive on molecular tests but negative by conventional methods is not known.

Recent epidemiologic studies based on CDI cases detected by Enzyme immunoassay (EIA) and culture positive samples have shown that approximately 25% of all CDI cases can be attributed to ward based transmission [4]. Whether additional cases that are detected by molecular methods (PCR) only contribute to hospital based transmission and has thus far been the undetected reservoir of infection, has never been formally studied. To examine this, we compared cases of *C. difficile* infection detected by PCR only (CYT negative) with cases detected both CYT and PCR in a hyperendemic pediatric population. We based assessment of transmission potential on bacterial carriage and genetic relatedness using the following,

1. Threshold cycle of detection as a surrogate marker for bacterial load and in turn greater risk of environmental contamination and;
2. Genetic relatedness using a highly discriminatory MLVA (multi-locus variable number tandem repeat analysis).

## Methods

Memorial Sloan Kettering Cancer Center (MSKCC) is a 470-bed tertiary care hospital in New York City with a 39 bed inpatient pediatric unit. Each year, there are approximately 1,500 pediatric admissions and 11,000 pediatric patient days annually. The average length of stay for pediatrics is 7.4 days. The pediatric day hospital (PDH) is a 36 bed facility for outpatient chemotherapy administration and outpatient evaluation and management with about 100 visits per day. Samples were collected from September 2010 until March 2011.

### Laboratory methods

All stool samples obtained from pediatric patients that tested positive for *C.difficile* were stored at −80°C within 24 hours of receipt in the lab. Patients with recurrent CDI or with duplicate specimens obtained within two weeks were excluded from the study.

#### Xpert *C. difficile* PCR

The assay was approved by the Food and Drug Administration (FDA) for the detection of *C. difficile* directly from stool specimens. The assay detects the toxin B gene within 1 hour with minimal hands-on time based on real-time PCR (Cepheid, Sunnyvale, CA). The Xpert ***C. difficile*** PCR (Xpert PCR) was performed according to the manufacturer's instructions and as previously described [5]. The cycle threshold (Ct) was defined as the number of PCR cycle required to generate a fluorescent signal above the background fluorescence [6]. It is a relative measure of the concentration of target gene in the PCR reaction.

#### Cytotoxin neutralization assay (CYT)

The CYT assay was performed as previously described. The assay detects the presence of the toxin B protein as measured through the presence of cytopathic effect in commercially available human lung fibroblast cell line (Diagnostics Hybrids, Athens, OH) [5].

#### *C. difficile* culture

*C. difficile* selective agar (CDSA; BD BBL, Sparks, MD) plates were reduced overnight in an anaerobic chamber prior to use. Stool sample was added to 500 µl of 100% ethanol, vortexed, and incubated at room temperature for 1 to 2 h. The solution was centrifuged at 1,200× *g* for 5 min, ethanol was removed, and the stool sample was inoculated onto reduced CDSA plates. The plates were incubated for 48 h under anaerobic conditions. Colonies resembling *C. difficile* (pale yellow to yellow) were sub cultured on sheep blood agar (SBA) plates, and their identity was further confirmed by Remel PRO disk (Thermo Fischer Scientific, Waltham, MA).

### MLVA

MLVA and *tcdC* sequencing were performed as previously described [7]. Resulting *tcdC* sequences were assigned genotypes by querying the PubMLST database (http://www.pubmlst.org/cdifficile). Minimum spanning trees of the MLVA data were generated using BioNumerics software v6.6 (Applied Maths, Austin TX). The summed tandem repeat difference (STRD) was used as coefficient for determining genetic distance. Based on validation studies performed in an outbreak setting comparing MLVA to REA (restriction enzyme analysis) and whole genome sequencing (WGS) [8] [9], STRD genetic relationships were defined as follows-

#### Outbreak

Strains with STRD≤2 are considered highly related and representative of an outbreak [9].

#### Genetically related

Isolates with genotypes having STRD greater than 2 but ≤10 are considered genetically related but not part of an outbreak [8], [9].

### Clinical data

*C. difficile* cases were defined by positive test (PCR or CYT assay). A retrospective chart review was conducted for all patients with positive specimens; clinical, laboratory and demographic data were extracted from the electronic MSKCC clinical information system. Demographic data included age and sex. Clinical data included underlying cancer, transplant type, inpatient stay and duration, presence or absence of diarrhea, previous CDI, and, when applicable, time to recurrence. Cases were defined as healthcare -associated (HA) or community acquired (CA) based on interval between admission and positive test result for CDI. An interval of ≥72 hours after admission was used to define HA cases of CDI. The MSKCC Internal Review Board reviewed the study and granted a HIPAA waiver of authorization.

### Statistical analysis

Statistical significance testing was performed using chi-squared tests of independence for categorical variables. The Student's t-test was used for mean age, assuming equal variances. The Wilcoxon Rank Sum test was used for median age.

## Results

A total of 54 new *C. difficile* cases occurred during the study period (September 2010 until March, 2011). Forty-seven samples were available for MLVA typing *C. difficile* could not be cultured from 5 frozen stool samples, 2 samples were lost during storage. Due to limited sample availability, 42/47 samples that were characterized by MLVA could be concurrently tested with CYT. These 42 samples were included in the final analysis.

For the 42 patients, the median age of the cohort was 11.2 years. 4/42 children were ≤2 years of age, 50% were females. Eighteen patients were allogeneic hematopoietic stem cell transplant (HSCT) recipients. Four patients had underlying ALL and five had neuroblastoma. 19/42 cases were healthcare associated and 38/42 (91%) of patients had diarrhea recorded at the time of testing. In three of the remaining four patients, the presence of diarrhea or change on bowel pattern could not be definitely ascertained due to presence of fecal incontinence, acute lower GI bleeding and chronic lower gastrointestinal GVHD. In one patient, testing was done for fever and abdominal pain that developed during stem cell infusion.

### MLVA and *tcdC* typing

**Figure 1:**
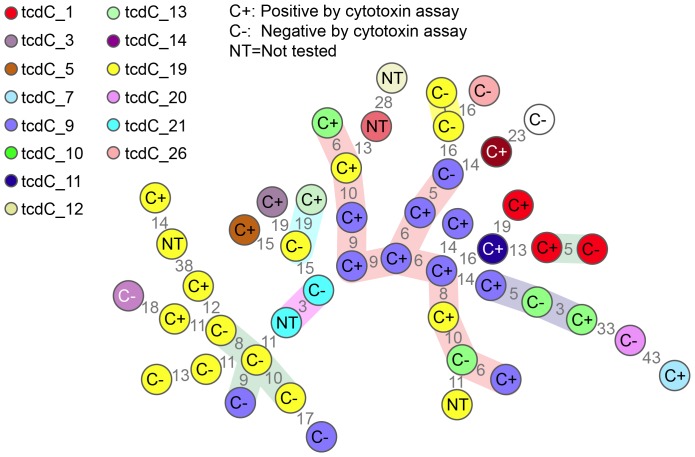
Minimum spanning tree of MLVA data from study isolates (n = 47). Letter symbol in the center of the circle represents the results for testing by cytotoxin assay for 42 samples included in the analysis (C + and C −). Five samples were not tested by CYT (NT). Each circle represents a distinct MLVA type and numbers between the circles represent the STRD [Summed tandem repeat difference]. Isolates with a STRD<10 are highlighted in colored clouds representing clusters (genetically related clonal complexes). *tcdC* sequencing is depicted by color coding within circles with *tcdC 1* (corresponding to NAP1) strain represented in red (reference in right corner).

The results of MLVA typing of the study cohort are shown in Figure 1 along with results of CYT testing. For 42 isolates that were tested by CYT and characterized by MLVA, 23 were positive by CYT and 25 were genetically related. Among the 25 strains that were genetically related, 13 were positive by CYT compared to 10/17 strains that were unrelated (p = 0.3).One dominant cluster accounted for almost half of all the related strains (n = 11); isolates within this cluster are related by MLVA but are not part of an outbreak (8). Rather, the strain corresponds to common genetic lineages as defined by *tcdC* genotyping and represent endemic disease in the hospital setting. 9/11 isolates in this dominant cluster were positive by CYT compared to 4/14 in non-dominant clusters (p = 0.01).

The *tcdC*-1 genotype (corresponding to Ribo type 027) was detected in four samples and was the third most common *tcdC* genotype isolated among our cohort. Isolates bearing the *tcdC*-19 genotype were the most common (32%) followed by *tcdC-9* genotype which was also highly prevalent (21%).

### Testing of PCR positive samples by CYT

**Figure 2:**
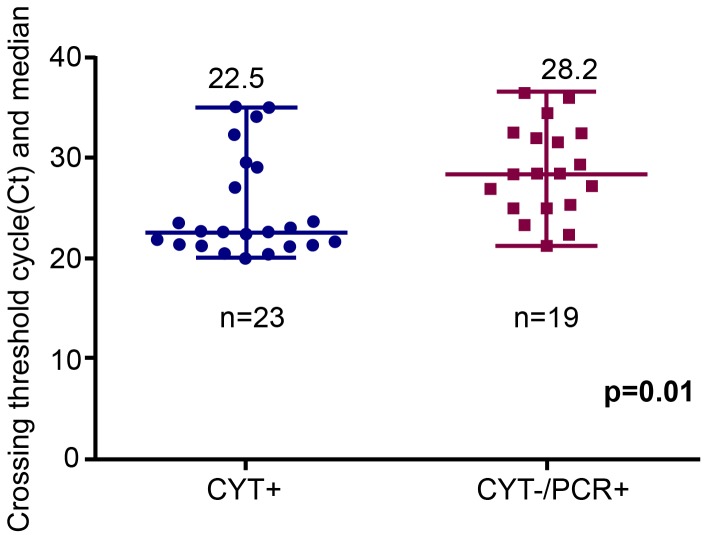
PCR threshold cycle value (Ct) for forty-two samples tested by Cytotoxin assay (CYT) and PCR.

Among 42 samples on which CYT was performed, 23 were found to be positive by CYT. For samples included in the present study on which both CYT and PCR were performed, we found that CYT −/PCR + cases had a higher median Ct value of detection compared to CYT+/PCR + cases (28.2 vs 22.5; p = 0.01; 95%CI 0.85 to 6.8) [Figure 2].

## Discussion

The introduction of highly sensitive diagnostic tests has led to identification of additional cases of many diseases with the potential for hospital transmission. This in turn has led to a need to estimate the relative contagiousness of the additional cases detected only by molecular methods. To explore this, we examined the relationship between detection method and genetic relatedness as determined by MLVA in a group of *C.difficile* isolates collected over a six month period in a closed population of children with cancer.

We found a significant relationship between low Ct value and concurrent positivity on cytotoxin assay. However, we did not detect any difference in genetic relatedness of isolates detected by the different testing methods. The only striking finding is the concomitant positivity on CYT among majority (9/11) of isolates belonging to the dominant endemic strain in the cohort. Our findings establish that cases detected by PCR only contribute towards hospital based transmission. However, dominant endemic clones are likely to be CYT positive, whether this is related to higher bacterial load or strain characteristics, needs to be addressed in future studies.

Children with cancer are a particularly suitable group for examination of the transmission dynamics of CDI. Similar to adults, cancer poses a substantial risk for CDI in children. The rate of CDI is sixteen times higher in children with cancer than other hospitalized children; children at highest risk include those with hematological cancer or those undergoing hematopoietic stem cell transplant [10], [11].Secondly, children with cancer may have diarrhea for multiple reasons and diagnostic tests that detect toxin gene (PCR) rather than toxin itself may detect colonization. For many years, testing and treating infants and children for CDI was discouraged because of recognition that asymptomatic colonization was common [12]–[16]. However, the appearance of the hyper virulent NAP1 strain among older children in the community led to a new appreciation of the potential role of this organism in the young, as did reports of its role as a pathogen among children with cancer and other chronic medical conditions. As a result, over the last decade there has been a renewed interest in examining the epidemiology of *C. difficile* in healthy and hospitalized children [14], [17]–[19].

At the same time as the apparent shift in epidemiology of CDI [20]–[22], PCR based detection of *C. difficile* has increasingly been adopted for diagnosis. The implementation of this technology carries uncertainty due to potential over-detection since the test cannot distinguish between colonization and true disease. Many centers find that PCR increases the detection of *C. difficile* by up to two fold and the incremental cases detected by PCR only, often are clinically mild [2]. Recent study by Curry et al examines transmission pattern from hospitalized patients with asymptomatic *C. difficile* colonization compared to those with CDI(using found specimens submitted for screening of VRE), their findings attributed 30% of incident cases to CDI patients, whereas 29% cases were associated with carriers. Although PCR was not used for screening, this study highlights the role of asymptomatic colonization with *C. difficile* in hospital based transmission [23].

Our data in a closed population of children with cancer supports that incremental new cases detected by real-time PCR are genetically related to endemic strains and likely contribute towards previously undetected transmission; however most cases caused by the dominant endemic strain in our cohort were detected using conventional methods. Our study has several limitations; we were unable to retrieve seven samples in the cohort and therefore may have missed an outbreak, although this seems unlikely as the cases were spread out in time and space. We had very few patients less than 24 months old [seven patients] and are unable to draw any meaningful conclusions about strain prevalence and CDI related outcomes in this group of infants. We used a highly discriminatory typing method- although our results were objective, unambiguous and reproducible, comparisons with other studies that have used a wide variety of molecular typing methods, had to made indirectly by correlation with REA, PCR ribotypes and *tcdC* genotypes. We do not think this limit the findings of our study as establishment of such genetic relationships has been corroborated by other studies [7], [24].Also, Xpert *C. difficile* assay is only FDA approved as a qualitative assay, its use as a semi-quantitative assay will need further validation. Ct values were derived from stool samples- volume loaded on swabs may have had an impact on the results, although processing of samples is done following standard protocol and our findings support that such bias is unlikely to have influenced the typing results. Finally, the epidemiology of CDI may be different in pediatric patients with cancer as compared to other hospitalized children due to frequent healthcare related exposure, underlying immunosuppression and greater antibiotic use.

In summary, in a closed population of children with cancer, we have found that additional *C. difficile* cases detected by PCR only are genetically related to endemic strains in the hospital and represent previously undetected transmission, although the dominant endemic clones are likely to be positive when tested using conventional methods.
